# Adsorption Behavior of Co^2+^, Ni^2+^, Sr^2+^, Cs^+^, and I^−^ by Corrosion Products α-FeOOH from Typical Metal Tanks

**DOI:** 10.3390/ma17112706

**Published:** 2024-06-03

**Authors:** Yingzhe Du, Lili Li, Yukun Yuan, Yufaning Yin, Genggeng Dai, Yaqing Ren, Shiying Li, Peng Lin

**Affiliations:** China Nuclear Power Technology Research Institute Co., Ltd., Shenzhen 518000, China; duyingzhe@126.com (Y.D.); lilili_mri@163.com (L.L.); yuanyukun@cgnpc.com.cn (Y.Y.); 18122062505@163.com (Y.Y.); genggengdai@126.com (G.D.); ryq1220@foxmail.com (Y.R.); mojinxinlsy@163.com (S.L.)

**Keywords:** Co^2+^, Ni^2+^, Sr^2+^, Cs^+^, I^−^, adsorption, α-FeOOH

## Abstract

Throughout the nuclear power production process, the disposal of radioactive waste has consistently raised concerns about environmental safety. When the metal tanks used for waste disposal are corroded, radionuclides seep into the groundwater environment and eventually into the biosphere, causing significant damage to the environment. Hence, investigating the adsorption behavior of radionuclides on the corrosion products of metal tanks used for waste disposal is an essential component of safety and evaluation protocols at disposal sites. In order to understand the adsorption behavior of important radionuclides ^60^Co, ^59^Ni, ^90^Sr, ^135^Cs and ^129^I on α-FeOOH, the influences of different pH values, contact time, temperature and ion concentration on the adsorption rate were studied. The adsorption mechanism was also discussed. It was revealed that the adsorption of key nuclides onto α-FeOOH is significantly influenced by both pH and temperature. This change in surface charge corresponds to alterations in the morphology of nuclide ions within the system, subsequently impacting the adsorption efficiency. Sodium ions (Na^+^) and chlorate ions (ClO_3_^−^) compete for coordination with nuclide ions, thereby exerting an additional influence on the adsorption process. The XPS analysis results demonstrate the formation of an internal coordination bond (Ni–O bond) between Ni^2+^ and iron oxide, which is adsorbed onto α-FeOOH.

## 1. Introduction

Due to the widespread use of nuclear energy worldwide, the management and disposal of hazardous spent fuel and radioactive waste have become significant environmental concerns [1]. Geological disposal is internationally acknowledged as the preferred approach for the long-term management of high level radioactive waste (HLW) [2,3,4,5]. In the disposal repository, multi physical barriers are designed to prevent the release of radionuclides, such as the HLW glass solidified body, the HLW disposal tank, the buffer/backfill material, and the surrounding rock of the repository. For the HLW disposal tank, it is mostly made by carbon steel, copper, stainless steels, titanium alloys or nickel alloys [6]. Within the multi-barrier system designed for the deep geological disposal of HLW, the metal container located near the buffer/backfill material serves as the initial barrier, isolating HLW from the biosphere. Its operational lifespan symbolizes the entire journey from geological disposal to the corrosion damage of HLW [7,8,9,10]. However, during the long-term waste treatment process, the HLW disposal tank may fail due to corrosion caused by groundwater infiltration into the storage. At this time, the corrosion layer of iron containers should be a key barrier to control and delay the migration of radionuclides [11]. When metal tanks used for waste disposal corrode, allowing local water to penetrate, radionuclides slowly dissolve from the waste matrix. They then migrate through the buffer layer and lithosphere via processes such as advection, dispersion, and diffusion, ultimately entering the biosphere. The radionuclides mitigation can lead to significant environmental harm [3]. Therefore, the investigating of radionuclide adsorption behavior onto corrosion products from metal tanks used in waste disposal is an essential component of safety and evaluation practices at disposal sites. Corrosion products commonly found in metal tanks include white rust (Fe(OH)_2_), magnetite (Fe_3_O_4_), goethite (α-FeOOH), and denser hematite (α-Fe_2_O_3_), with their formation progressing from the inner to the outer layers [12]. α-FeOOH, being one of the most stable iron oxides, has received considerable attention in the realm of soil and water quality remediation [13,14]. α-FeOOH is the most common iron hydroxide found in nature. It possesses a large specific surface area, numerous active sites, variable surface charges, and is relatively easy to synthesize in a laboratory [15,16]. Furthermore, it exhibits a particularly high affinity for a wide range of anions [17,18] and cations [1,19,20].

Radionuclides as ^60^Co, ^59^Ni, ^90^Sr, ^135^Cs, ^129^I, ^233^U, and ^99^Tc, characterized by their long half-lives and ease of diffusion mechanisms, can infiltrate organisms through water, soil, animals, and plants, persist for extended periods, and pose a threat to human health as they enter the food chain [21,22]. Co^2+^ can induce neurotoxicological diseases and genotoxicity in humans, and chronic exposure can increase the risk of cancer [23]. The radionuclide ^63^Ni (with a half-life of 96 years) is a significant byproduct of neutron activation in reactor materials, and the investigation of Ni^2+^ is essential for assessing the behavior of ^63^Ni in the environment [24]. Sr^2+^ is a highly toxic radionuclide that readily infiltrates the skeletal systems of organisms, leading to radiation damage [25]. Cs^+^ and I^−^ nuclides are pervasive in the Earth’s environment, entering the human body through the food chain and posing significant threats to the biosphere [26]. Previous studies have shown that the adsorption of Ni^2+^ by α-FeOOH was positively responsive to the increase in the amount, time and temperature of the adsorbent. The adsorption behavior of Ni^2+^ on the α-FeOOH (0 1 0) plane simulated by Molecular Dynamics (MD) shows that NiCl_2_ concentration is highest in the 30–40 Å region [27]. At low pH, the adsorption of Co^2+^ by α-FeOOH is dependent on ionic strength and foreign ions, while at high pH, the adsorption is independent of ionic strength and foreign ions. In addition, the presence of FA promotes the adsorption of Co^2+^ to α-FeOOH at low pH, but inhibits the adsorption of Co^2+^ to α-FeOOH at high pH [15]. The EXAFS data showed that the inner-sphere complexes with goethite at alkaline conditions and the outer-sphere complexes at moderate pH [28,29]. Based on the analysis of ion exchange mechanism, the higher the pH value, the higher the adsorption efficiency of iron oxides α-Fe_2_O_3_ and Fe_3_O_4_ on ^60^Co, ^90^Sr and ^137^Cs. Regardless of oxidation properties, the absorption characteristics of ^60^Co are the best, and the absorption characteristics of ^137^Cs are the worst, forming an arrangement of ^60^Co > ^90^Sr > ^137^Cs [30].

In conclusion, the adsorption of radionuclides by iron oxides has great potential. In addition, there are few studies on the adsorption behavior of nuclides ^60^Co, ^59^Ni, ^90^Sr, ^135^Cs and ^129^I based on corrosion products from HLW disposal tanks. In this work, the adsorption behavior of Co^2+^, Ni^2+^, Sr^2+^, Cs^+^ and I^−^ in α-FeOOH corrosion products of typical metal cans was preliminarily investigated. We achieved this by simulating the groundwater environment, exploring the impact of factors such as pH, temperature, and ionic strength on the adsorption behavior, and discussing the potential adsorption mechanisms. In this paper, we collectively refer to these five ions as nuclide ions.

## 2. Adsorption Experiment

### 2.1. Materials Preparation

KOH and Na_2_CO_3_ were purchased from Tianjin Zhiyuan Chemical Reagent Co., Ltd., Tianjin, China. NaOH was purchased from Beijing Oriental Shibo Fine Chemical Co., Ltd., Beijing, China. HNO_3_ was purchased from Tianjin Kemi Ou Chemical Reagent Co., Ltd., Tianjin, China. Co(NO_3_)_2_·6H_2_O was purchased from Innochem (Beijing) Technology Co., Ltd., Beijing, China. Ni(NO_3_)_2_·6H_2_O and CsNO_3_ were purchased from Shanghai Aladdin Biochemical Technology Co., Ltd., Shanghai, China. Fe(NO_3_)_3_, Sr(NO_3_)_2_, KI and NaClO_4_ were purchased from Sinopharm Chemical Reagent Co., Ltd., Shanghai, China. All of these chemicals were analytical pure. In addition, 18.2 MΩ deionized ultra-pure water was used for preparing solutions. The calculated dosages of the prepared reserve solution are shown in Table 1. The α-FeOOH sample was prepared using the precipitation-aging method [15]. KOH (5 mg∙L^−1^, 180 mL) and Fe(NO_3_)_3_ (1 mol∙L^−1^, 100 mL) were mixed in a polypropylene (PP) plastic bottle and diluted with deionized water to a total volume of 2 L. After heating the mixture to 70 °C, the plastic bottle was sealed. After three days of the aging reaction, the mixture was cooled to room temperature and then centrifuged. The solid phase was rinsed with deionized water, dried at 60 °C for 24 h, ground, and passed through a 200-mesh sieve to obtain FeOOH powder.

### 2.2. Adsorption Experiment for α-FeOOH

In all experimental batches, the pH of α-FeOOH suspension and the reserve solution containing key nuclides were adjusted using appropriate HNO_3_ and NaOH/Na_2_CO_3_ solutions, respectively. The two solutions were then mixed in a centrifuge tube, following a solid–liquid ratio (2 g∙L^−1^) and oscillated on a constant temperature oscillator. The concentrations of Co^2+^ and Ni^2+^ were measured by ICP-OES (Horiba JY2000-2, Kyoto, Japan), and the wavelength of the spectral lines selected for the detection of Co and Ni were 228.62 nm (Co) and 231.60 nm (Ni), respectively. Standard curves, ranging from 0 to 5 mg/L, were established using standard solutions containing specified concentration of(0 mg/L, 1 mg/L, 2 mg/L and 5 mg/L) corresponding element. The samples to be measured were diluted different times so that their concentration fell within the range of standard curve. The concentration of Sr^2+^, Cs^+^ and I^−^ were measured using internal standard method in ICP-MS (Thermo X7, Waltham, MA, USA), selecting ^115^In as the internal standard. The isotopes of these elements selected for determination were ^88^Sr, ^133^Cs, and ^127^I. The concentration range of the standard curve is 0–100 ppb (the concentration selection points of the standard curve are 0 μg/L, 0.1 μg/L, 0.5 μg/L, 1 μg/L, 5 μg/L, 10 μg/L, 50 μg/L and 100 μg/L). X-ray photoelectron spectra (XPS) were obtained using an ESCALAB 250Xi (Thermo Scientific, Waltham, MA, USA) spectrometer equipped with an Al Kα source (1486.8 eV). XPS spectra were calibrated by setting the major C–C peak of adventitious carbon to 284.8 eV and fitted using the Shirley algorithm background. The crystal structures of α-FeOOH were characterized by powder X-ray diffraction (XRD, Bruker D8 Advance, Billerica, MA, USA, Cu Kα X-ray source, λ = 1.5406 Å, step size 0.02°). The scanning rate was 2°/min from 15° to 70° in 2θ. Scanning electron microscopy (SEM) was performed on a HITACHI Regulus8100 instrument (Tokyo, Japan). The surface area of α-FeOOH measurements were conducted using Brunauer–Emmett–Teller (BET) method by the instrument ASAP 2020 (Micrometrics, Norcross, GA, USA). The Malvern Zetasizer Nano ZS90 (Malvern, UK) zeta potential analyzer was used to determine the zeta potential of α-FeOOH.

With the variation of concentration before and after adsorption, the adsorption capacity and adsorption distribution coefficient *K_d_* of α-FeOOH for nuclide ions were calculated by the following formula [7]:

(1) $$q_{e}=\frac{(C_{0}-C_{e})\times V}{m}$$

(2) $$K_{d}=\frac{C_{0}-C_{e}}{C_{e}}\times \frac{V}{m}$$

where *C*_0_ (mg∙L^−1^) and *C_e_* (mg∙L^−1^) is the initial and after adsorption concentration of key nuclides in sample, respectively, *q_e_* (mg∙g^−1^) is the adsorption capacity of α-FeOOH for key nuclide ions, *K_d_* is the distribution coefficient of nuclide ions in the solid–liquid phase, *V* (L) is the volume of the suspension and *m* (g) is the mass of α-FeOOH.

### 2.3. Preliminary Experiment

The suspension of iron oxide adsorbent and the reserve solution containing nuclide ions are adjusted to the specified pH using an appropriate amount of HNO_3_ and NaOH/Na_2_CO_3_ solution. Two solutions were mixed in a centrifugal tube in accordance with a specified solid–liquid ratio. The mixture was subsequently subjected to oscillation on a constant temperature oscillator to ensure a proper mixing and the homogenization of the solution. Samples were taken at a certain time, and pH of the system was recorded within 10 min after mixing and after the experiment. One group of experiments were conducted for Co^2+^, Ni^2+^, Sr^2+^, Cs^+^ and I^−^, and each group of experiments contained three parallel samples. The results show that the removal rate of Co^2+^ and Ni^2+^ by iron oxide is moderate (35~40%) at the initial concentration of 5 ppm and near neutral pH. The results not only ensure the accuracy of ICP-OES measurement, but also reflect the change of adsorption behavior in a wide range of pH. Under the same conditions, Sr^2+^, Cs^+^ and I^−^ have relatively poor adsorption performance of the nuclide ion, and the instrument error significantly impacts the accuracy of concentration measurement. Therefore, the initial concentration of these three nuclides is reduced to 1 ppm later in this work, and ICP-MS with higher measurement accuracy was used to determine the concentration of related adsorption samples. In order to better explore the adsorption effect of iron oxides on the five kinds of nuclides under different conditions, the reaction conditions selected after the pre-experiment are shown in Table 2.

### 2.4. Adsorption Thermodynamics

The calculation of thermodynamic parameters and the analysis of apparent changes in enthalpy (Δ*H*), entropy (Δ*S*), and Gibbs free energy (∆*G*) during the adsorption process are crucial for assessing the feasibility and spontaneity of the process. The thermodynamic properties of the target nuclide ions, namely entropy, enthalpy, and Gibbs free energy (Δ*S*, Δ*H*, ∆*G*), can be determined by calculating the distribution coefficients at various temperatures and substituting them into the relevant equation. This methodology enables the assessment of temperature effect on the adsorption characteristics of crucial nuclide ions by the corrosion products of typical metal tanks. The formula for thermodynamic calculations is presented below [31]:

(3) $$\ln K_{d}=\frac{\Delta S}{R}-\frac{\Delta H}{RT}$$

(4) ΔG=ΔH−T×ΔS

where *K_d_* is the distribution coefficient (mL∙g^−1^); *T* is the absolute temperature (K); *R* is the ideal gas constant (8.314 J·mol^−1^·K^−1^); ∆*S* is the change in entropy (J·mol^−1^·K^−1^); ∆*H* is the enthalpy change(J·mol^−1^); ∆*G* is the Gibbs free energy change (J·mol^−1^).

### 2.5. Adsorption Kinetics

In this work, kinetic models were used to further analyze the adsorption of key nuclides on α-FeOOH The equilibrium adsorption capacity (*q_e_*) calculated by the experimental model or by the kinetic model were compared to verify the reliability of experimental results. A pseudo-first-order kinetic model and a pseudo-second-order kinetic model were used to fit the adsorption results of five nuclides, respectively. The two kinetic models are described by Equations (5) and (6) [32]:

(1) pseudo-first-order kinetic model:

(5) $$\log(q_{e}-q_{t})=\log q_{e}-\frac{k_{1}}{2.303}\times t$$

(2) pseudo-second-order kinetic model:

(6) $$\frac{t}{q_{t}}=\frac{1}{k_{2}q_{e}^{2}}+\frac{t}{q_{e}}$$

where *q_e_* and *q_t_* are adsorption amounts at equilibrium and time *t*, respectively (mg∙g^−1^). *k*_1_ (min^−1^) and *k*_2_ (g∙mg^−1^∙min^−1^) are rate constants of pseudo-first-order and pseudo-second-order kinetic models, respectively.

## 3. Results

In this study, α-FeOOH, a typical corrosion product of metal tanks, was selected as the adsorbent to investigate the adsorption behavior of key nuclide ions under various influencing factors. The influences of environmental factors, including contact time, pH, temperature, and ionic strength, on the adsorption efficiency of key nuclides Co^2+^, Ni^2+^, Sr^2+^, Cs^+^ and I^−^ were investigated. Different kinetic models were used to analyze the adsorption process.

### 3.1. Characterization

**Figure 1:**
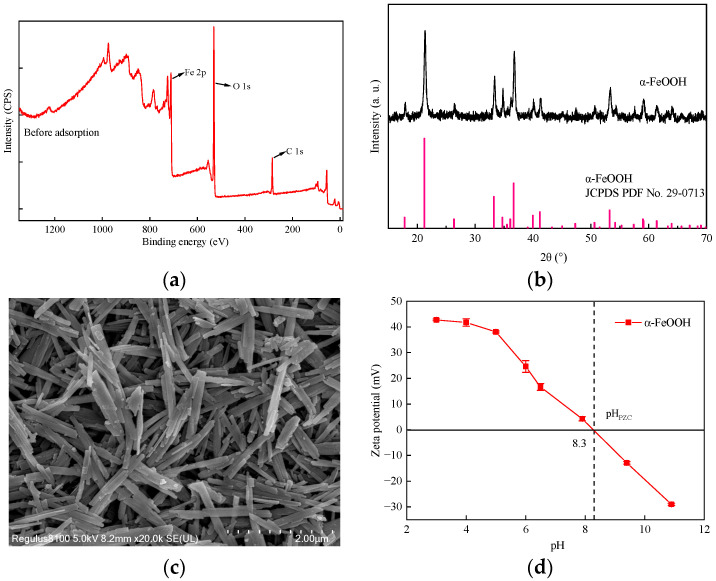
Characterization diagrams of α-FeOOH. (**a**) XPS survey spectrum of α-FeOOH; (**b**) XRD patterns of α-FeOOH; (**c**) SEM image; (**d**) zeta potential in function of pH.

The surface chemical composition and bonding information of the adsorbent α-FeOOH obtained by XPS are presented in Figure 1a. The spectrum shows that the adsorbent consists mainly of Fe and O. No N (binding energy of about 400.0 eV) [33] and K (binding energy of about 292.9–295.7 eV) [34] were identified, indicating that nitrate and potassium ions from raw materials were effectively removed by the cleaning process.

Figure 1b shows the XRD pattern of α-FeOOH. It can be seen that the α-FeOOH before adsorption well matches the standard card of goethite (JCPDS:29-0713). The correspondence of the position and intensity of the peaks proves that the synthesized α-FeOOH is well crystallized. The BET specific surface area of α-FeOOH is measured to be 39.4386 m^2^∙g^−1^. The SEM image in Figure 1c, further prove that α-FeOOH of goethite has been successfully prepared [35]. Figure 1d presents the measured zeta potential of iron oxide α-FeOOH in function of pH. The point of zero charge (PZC) (pH corresponding to zero zeta potential) of the α-FeOOH particles was determined to be 8.3.

**Figure 2:**
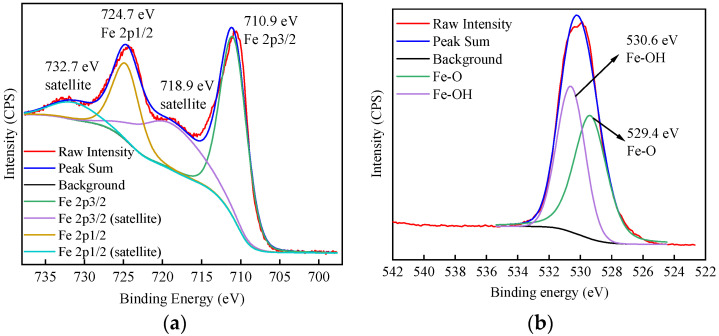

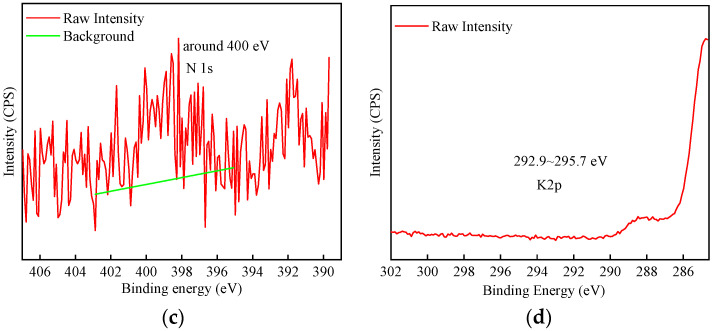
High-resolution spectrum of α-FeOOH. (**a**) Fe 2p; (**b**) O 1s; (**c**) N 1s; (**d**) K 2p.

Figure 2a shows the high-resolution Fe 2p spectrum of α-FeOOH before adsorption. The Fe 2p3/2 peak at 710.9 eV and its associated satellite peak at 718.9 eV, as well as the Fe 2p1/2 peak at 724.7 eV and its associated satellite peak at 732.7 eV, can be observed [36]. The results suggest that the valence state of Fe in α-FeOOH is Fe(III) [37]. Figure 2b shows the high-resolution O 1s spectrum of α-FeOOH. The peak at 529.4 eV is assigned to Fe-O, and the peak at 530.6 eV is assigned to Fe-OH [38,39]. The curve fitting result supports the presence of Fe-O and Fe-OH bonds in α-FeOOH, in line with its chemical composition. Figure 2c,d showed the N 1s and K 2p spectra of α-FeOOH, respectively. The absence of N 1s (around 400 eV) [40] and K 2p (292.9~295.7 eV) [41] peaks confirms that the sample does not contain any N and K elements.

### 3.2. Effect of Adsorption Time

**Figure 3:**
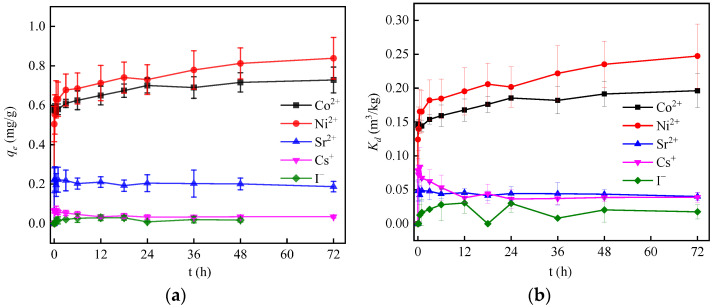
At pH 6.5~7.5, temperature 28 °C and NaClO_4_ 0 mmol/L, influence of contact time on adsorption effect. (**a**) *q_e_*; (**b**) *K_d_*.

Adsorption equilibrium time is one of the important parameters to evaluate the practicability of adsorbents. The influences of adsorption time on the adsorption of nuclide ions on α-FeOOH was investigated by controlling other conditions obtained in the preliminary experiment (pH 6.5~7.5, temperature 28 °C and NaClO_4_ 0 mmol/L). Figure 3 shows the adsorption kinetics curves of α-FeOOH for Co^2+^, Ni^2+^, Sr^2+^, Cs^+^ and I^−^. Experimental data show that in the early stage of adsorption, the adsorption rate of Co^2+^ and Ni^2+^ nuclides is relatively high, which may be due to the large number of adsorption active sites on α-FeOOH in the early stage. Subsequently, with the reduction in adsorption sites, the adsorption rate gradually flattens and the adsorption reaches equilibrium [42]. As shown in Table 3, the adsorption capacity of Co^2+^ and Ni^2+^ is positively correlated with time and is dependent on the number of active sites available on the adsorbent.

In Figure 3a, the initial adsorption capacity of Sr^2+^ and Cs^+^ increases during the first 1 h and then decreases, indicating that the adsorption of Sr^2+^ and Cs^+^ by α-FeOOH followed a pattern of initial adsorption and desorption resolution. In addition, the adsorption kinetic curve of α-FeOOH adsorption of I^−^ shows that the adsorption reaches equilibrium after 12 h.

### 3.3. Adsorption Kinetics Study

In this work, α-FeOOH adsorption kinetic of nuclide ions was studied under the conditions of adsorption time 72 h, pH 6.5~7.5, temperature 28 °C and NaClO_4_ 0 mmol/L. The interaction force of α-FeOOH adsorption on Sr^2+^ and Cs^+^ is weak [30], and the pH of the solution system will be slightly reduced during the adsorption process: Co^2+^ pH 7.0→pH 6.9; Ni^2+^ pH 7.2→pH 6.9; Sr^2+^ pH 7.4→pH 7.0; Cs^+^ pH 7.5→pH 7.3; I^−^ pH 7.6→pH 7.2. Changes in the pH of the solution tend to cause desorption of these weakly adsorbed nuclide ions. Due to the initial adsorption and desorption phenomenon caused by Sr^2+^ and Cs^+^, the fitting using the pseudo-first-order kinetic model and the pseudo-second-order kinetic model is not ideal, meaning that the obtained fitting parameters are unreasonable, thus are not listed.

**Figure 4:**
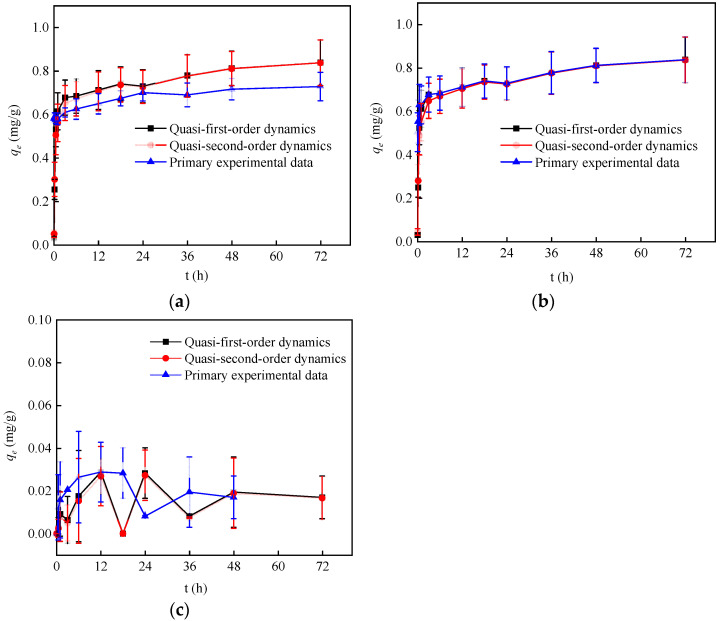
Kinetic fitting curves of α-FeOOH adsorption of Co^2+^, Ni^2+^ and I^−^. (**a**) Co^2+^; (**b**) Ni^2+^; (**c**) I^−^.

The change of the adsorption capacity of α-FeOOH for Co^2+^, Ni^2+^ and I^−^ over time is shown in Figure 4. It can also be seen from Figure 4 that both the pseudo-first-order kinetic model and the pseudo-second-order kinetic model fit the primary experimental data well. The equilibrium adsorption capacity (*q_e_*) of α-FeOOH for Co^2+^, Ni^2+^ and I^−^ are 0.674 mg·g^−1^, 0.743 mg∙g^−1^ and 0.0295 mg·g^−1^, respectively, close to the experimental results. Table 4 shows the fitting kinetic parameters of the adsorption of Co^2+^, Ni^2+^ and I^−^ by α-FeOOH. As can be seen from Table 4, compared with the pseudo-first-order model, the correlation coefficient R^2^ of the pseudo-second-order kinetic equation is closer to one. This indicated that the adsorption of Co^2+^, Ni^2+^ by α-FeOOH is more consistent with the pseudo-second-order kinetic model. This indicates that α-FeOOH adsorption of Co^2+^, Ni^2+^ may be chemisorption.

### 3.4. Effect of pH

**Figure 5:**
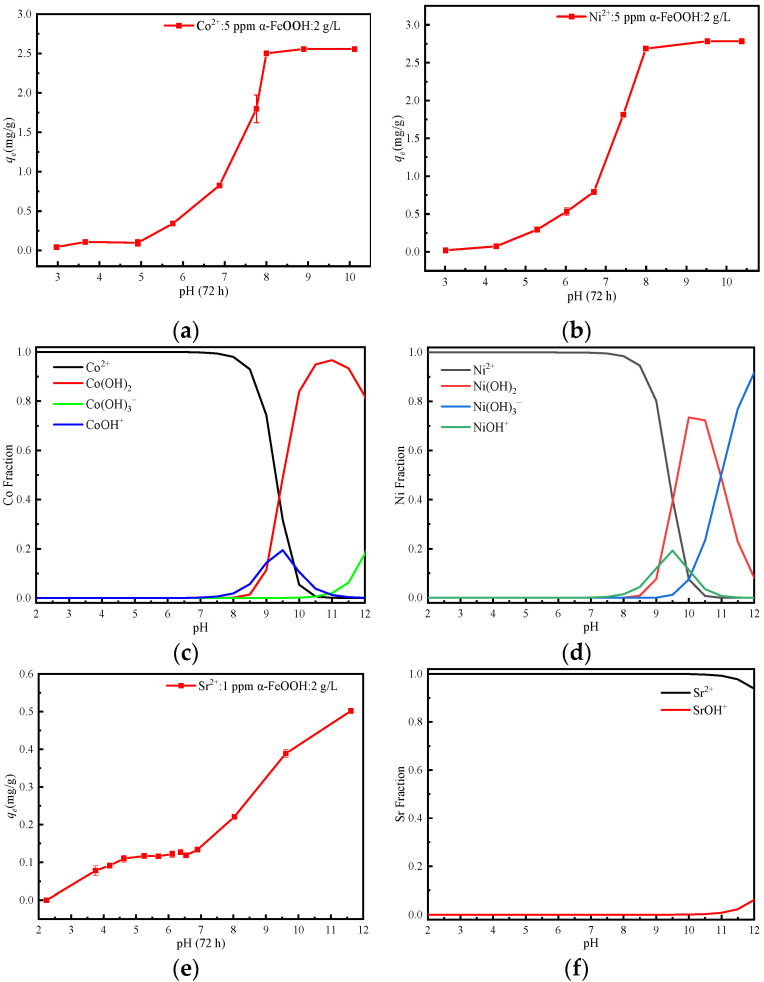

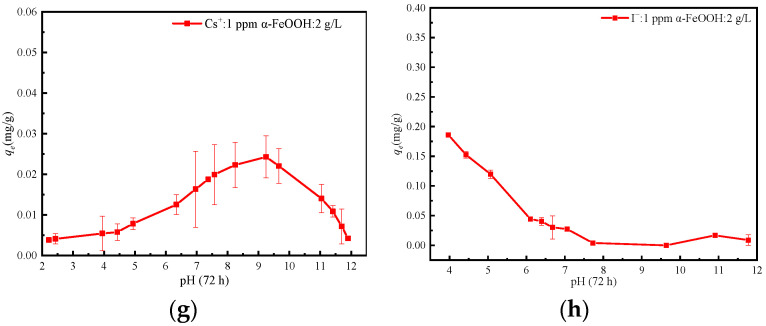
Effect of pH on the adsorption of (**a**) Co^2+^, (**b**) Ni^2+^, (**e**) Sr^2+^, (**g**) Cs^+^, and (**h**) I^−^ by α-FeOOH and probability distribution graph of (**c**) Co^2+^, (**d**) Ni^2+^, and (**f**) Sr^2+^.

The influence of pH on the adsorption behavior of Co^2+^, Ni^2+^, Sr^2+^, Cs^+^, and I^−^ by α-FeOOH is shown in Figure 5. In this work, the species state distribution of five ions at different pH was calculated by using the species state analysis simulation software Visual MINTEQ 3.1. As can be seen from Figure 5a,b the adsorption capacity of α-FeOOH for Co^2+^ and Ni^2+^ increases with the increase in pH in the range of 3~8. In Figure 5e, the adsorption capacity of α-FeOOH for Sr^2+^ increases with increasing pH. The effect of pH on the adsorption is related to the surface charge properties of iron oxide at different pH conditions and the distribution of the two nuclide ions [43,44,45,46]. Figure 5c,d show that in acidic and near-neutral environments, nuclides exist primarily in the form of Co^2+^ and Ni^2+^. Figure 5f shows that Sr^2+^ exists predominantly as Sr^2+^ when the solution pH < 10.0. When pH < pH_PZC_(8.3), the surface of α-FeOOH carries a positive charge, leading to electrostatic repulsion that hinders the adsorption of Co^2+^, Ni^2+^, Sr^2+^, and Cs^+^. However, at pH > pH_PZC_(8.3), the surface charge of α-FeOOH shifts from positive to negative. Meanwhile, Co^2+^ and Ni^2+^ undergo partial hydrolysis to form MOH^+^, facilitating the formation of strong internal coordination complexes with iron oxide. This results in a rapid increase in adsorption capacity [23]. At higher pH levels, Co(OH)_2_ and Ni(OH)_2_ colloids form in the system and easily adhere to the adsorption material’s surface, achieving a nuclide ion removal rate of up to 100%. Outer-sphere surface complexation and/or ion exchange were the main mechanisms of Co^2+^ adsorption on α-FeOOH at low pH values, whereas innersphere surface complexation was the main adsorption mechanism at high pH values [15]. When the pH > 11.0, Sr^2+^ gradually hydrolyzes to form SrOH^+^, enhancing adsorption via electrostatic attraction and further increasing adsorption capacity [1].

Figure 5g illustrates that the adsorption capacity of α-FeOOH for Cs^+^ initially rises and subsequently decreases with increasing pH. Under acidic conditions, the surface of α-FeOOH becomes positively charged, resulting in a significantly reduced adsorption capacity for Cs^+^ due to electrostatic repulsion. At pH levels above pH_PZC_(8.3), the surface of α-FeOOH becomes negatively charged, facilitating a further enhancement of adsorption capacity, with maximum adsorption occurring at pH = 9. With a further increase in pH, the adsorption capacity began to decline, possibly due to the increased introduction of Na^+^ while adjusting the high pH solution. This decrease resulted from the weak electrostatic interaction between Cs^+^ and α-FeOOH and the significant reduction in adsorption capacity of Cs^+^ due to the introduction of other competitive cations with a certain concentration in the system [37]. At low pH, I^−^ is unstable and prone to oxidation by HNO_3_, so pH > 4.0 is selected for research. As illustrated in Figure 5h, the adsorption capacity of I^−^ gradually decreases with the increase in pH. In weakly acidic environments, the surface of α-FeOOH becomes positively charged, thereby enhancing the adsorption. As pH increases, the positive surface charge of α-FeOOH decreases, leading to a gradual reduction in the adsorption capacity of I^−^. When the pH exceeds pH_PZC_, the surface charge of α-FeOOH shifts from positive to negative, leading to a substantial decrease in the adsorption capacity of I^−^.

### 3.5. Effect of Temperature and Thermodynamic Analysis

**Figure 6:**
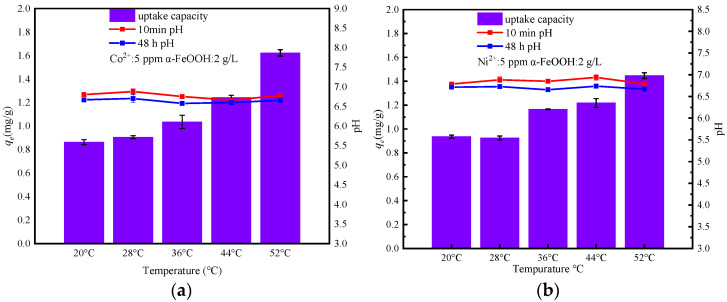

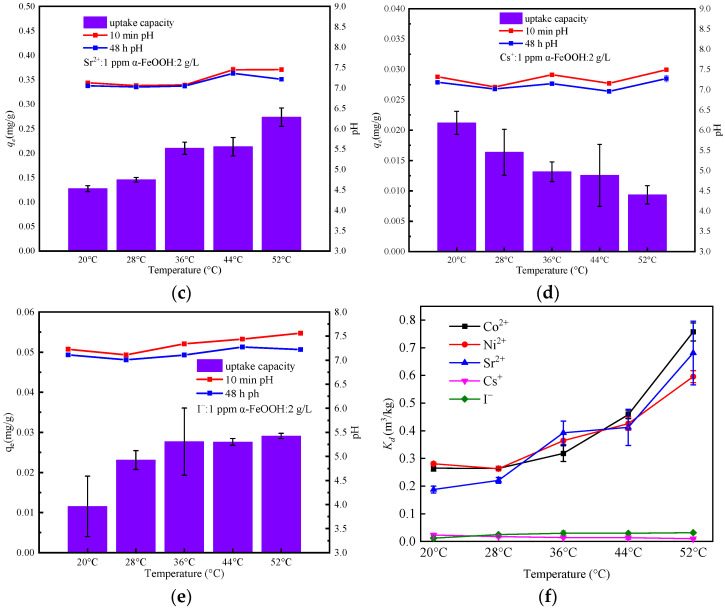
Effect of temperature on the adsorption of (**a**) Co^2+^, (**b**) Ni^2+^, (**c**) Sr^2+^, (**d**) Cs^+^ and (**e**) I^−^ by α-FeOOH. (**f**) Effect of temperature on *K_d_*.

The current experimental study aimed to explore the influence of different temperatures (20 °C, 28 °C, 36 °C, 44 °C, and 52 °C) on the adsorption effects. Analysis of Figure 6a–c,e revealed significant effects of temperature elevation on the adsorption of Co^2+^, Ni^2+^, Sr^2+^ and I^−^ by α-FeOOH. The experimental results indicate a gradual increase in the adsorption capacity (*q_e_*) of α-FeOOH for Co^2+^, Ni^2+^, Sr^2+^ and I^−^ as the temperature increases from 20 °C to 52 °C. This increase is accompanied by a corresponding rise in *K_d_* values. In addition, it can be seen from Table 5 that Δ*S* > 0 and Δ*H* > 0 in the adsorption process of these four nuclide ions indicate that α-FeOOH adsorption of Co^2+^, Ni^2+^, Sr^2+^ and I^−^ is a process of entropy increase and heat absorption, and temperature increase is conducive to adsorption. In the study temperature range, Δ*G* is less than 0, indicating that α-FeOOH adsorption of Co^2+^, Ni^2+^, Sr^2+^ and I^−^ is a spontaneous reaction, and Δ*G* becomes more negative with the increase in temperature, which further indicates that high temperature is conducive to adsorption.

Figure 6d indicates a gradual decrease in the adsorption capacity of α-FeOOH for Cs^+^ as the temperature increases. The *K_d_* values corresponding to different temperature conditions are consistently low, ranging from 0.023 to 0.010 m^3^∙kg^−1^. Additionally, the thermodynamic analysis of Table 5 reveals that the adsorption of Cs^+^ by α-FeOOH is an exothermic process, accompanied by a reduction in entropy. Furthermore, as the temperature rises, Δ*G* also increases, further indicating that low temperature is more conducive to the adsorption [47,48].

### 3.6. Effect of Ionic Strength

**Figure 7:**
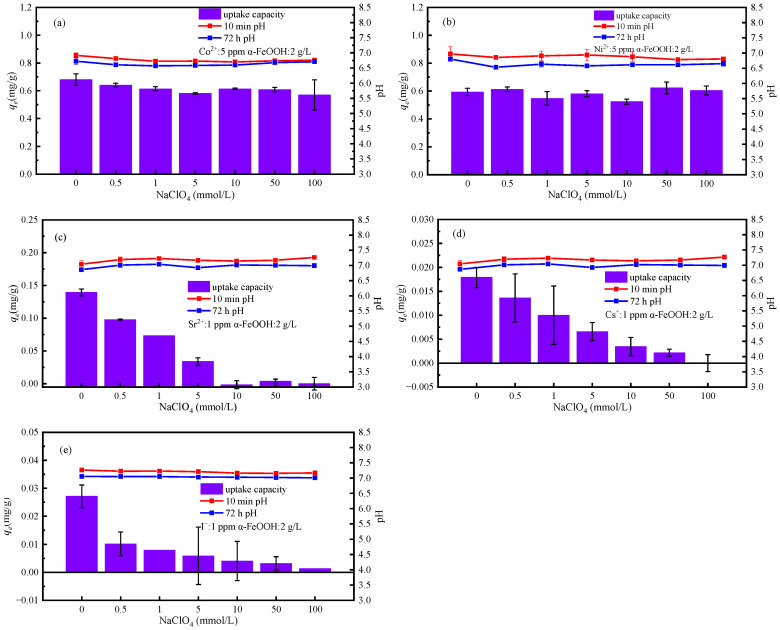
Effect of ion strength on the adsorption of (**a**) Co^2+^, (**b**) Ni^2+^, (**c**) Sr^2+^, (**d**) Cs^+^ and (**e**) I^−^ by α-FeOOH.

This study focuses on investigating the impact of the ClO_4_^−^ anion present in groundwater on adsorption. Nuclide ion solutions with varying concentrations of NaClO_4_ were individually mixed with adsorbent suspensions. The pH of both solutions was pre-adjusted to approximately 6.5~7.5. The pH levels were recorded at 10 min and 72 h. As depicted in Figure 7a,b the adsorption capacity *q_e_* of Co^2+^ and Ni^2+^ remains relatively constant at around 0.60 mg∙g^−1^ with increasing ion concentration, indicating that the adsorption performance of α-FeOOH for Ni^2+^ and Co^2+^ is minimally affected by ion strength. At pH = 7.0, Ni^2+^ and Co^2+^ form relatively stable inner coordination complexes on the surface of α-FeOOH. Under these conditions, competitive cations cannot displace the nuclide ions through outer coordination. Consequently, the increase in ionic strength has no significant impact on the adsorption capacity [38]. Figure 7c–e demonstrate a gradual reduction in the adsorption performance of α-FeOOH for Sr^2+^, Cs^+^ and I^−^ as ionic strength increases. The inclusion of a certain concentration of supporting electrolyte significantly inhibits the adsorption capacity of Sr^2+^, Cs^+^ and I^−^. This inhibition can be attributed to the weak force between the nuclide ions and α-FeOOH via outer layer coordination, allowing for the replacement of nuclide ions by competing coordination with negative ions and cations present in the electrolyte solution (Na^+^ competes with Sr^2+^, Cs^+^; ClO_4_^−^ competes with I^−^) [39]. Consequently, at high concentrations of NaClO_4_, the adsorption capacity of Sr^2+^, Cs^+^ and I^−^ approaches zero.

### 3.7. Adsorption Mechanism

**Figure 8:**
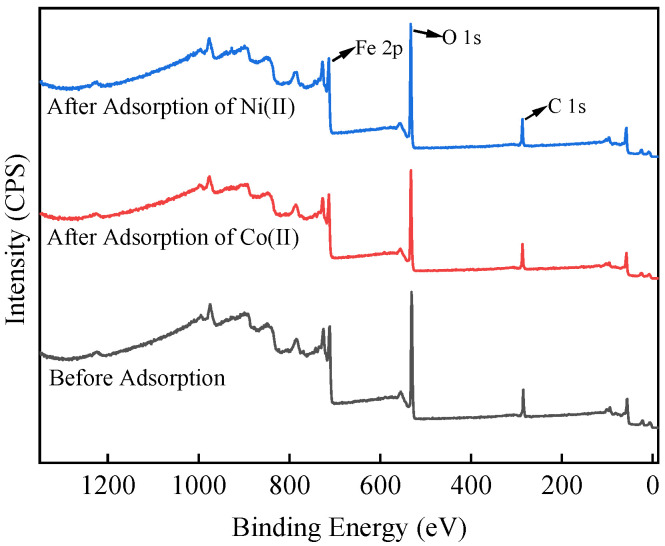
XPS full spectrum comparison of α-FeOOH before and after adsorption of Co^2+^ and Ni^2+^.

**Figure 9:**
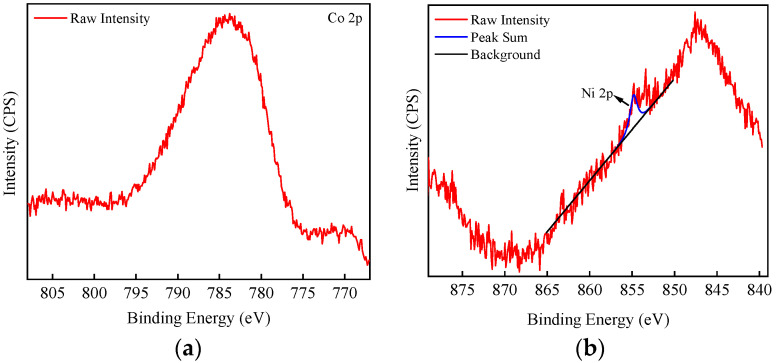
High-resolution spectrum of α-FeOOH after adsorption. (**a**) Co 2p; (**b**) Ni 2p.

In this study, we analyzed the surface chemical composition and bonding information of iron oxides before and after the adsorption of nuclide ions using a photoelectron spectrometer (XPS). The main objective was to investigate the mechanism of the adsorption effect between α-FeOOH and the nuclide ions. Figure 8 illustrates the XPS full spectrum comparison before and after α-FeOOH adsorption of Co^2+^ and Ni^2+^. The two full spectrums of α-FeOOH showed no remarkable difference. Subsequently, Figure 9a presents the high-resolution spectrum of Co 2p after α-FeOOH adsorption of Co^2+^. Generally, the binding energy of Co^2+^ is 781 eV [49,50]. However, in this spectrum, the peak is located at 784 eV, in good agreement with the Auger electron spectral line peak of Fe, indicating that the presence of a large amount of Fe in the adsorbent material determines the characteristic peak rather than the adsorbed Co. Therefore, the characteristic peak of Co cannot be definitively determined at this stage. Furthermore, Figure 9b demonstrates the high-resolution spectrum of Ni 2p after α-FeOOH adsorption of Ni^2+^; the peak at 847 eV is the Fe 2s characteristic peak of iron oxides [51]. In Figure 9b, the 854.4 eV peak corresponds to the Ni^2+^-O peak of nickel oxide (NiO), consistent with the binding energy of Ni-O bond reported in the literature [52,53]. This observation indicates that Ni^2+^ interacts strongly with oxygen on the surface of α-FeOOH. These findings suggest that α-FeOOH may bind to Ni^2+^ through internal coordination and that the chemical valence state of Ni remains unchanged during the adsorption process.

**Figure 10:**
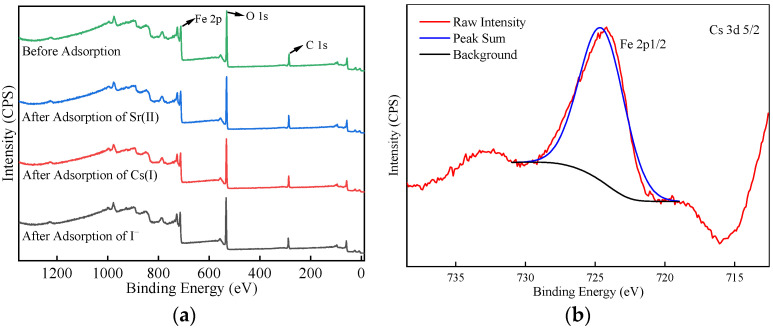
XPS full spectrum of α-FeOOH before and after adsorption of Sr^2+^, Cs^+^ and I^−^. (**a**) XPS images before and after adsorption; (**b**) Cs 3d.

Figure 10a depicts the full XPS spectrum before and after the adsorption of Sr^2+^, Cs^+^, and I^−^ on α-FeOOH. No evident difference is observed between the full spectra of α-FeOOH before and after adsorption. Figure 10b displays the Cs 3d spectrum after the adsorption of Cs^+^ on α-FeOOH. The characteristic peak of Cs 3d5/2 is at 724.8 eV, while the satellite peak of Fe (Fe 2p1/2) is located at 724 eV [20]. The presence of a substantial amount of Fe^3+^ in the sample interferes with the identification of Cs 3d characteristic peaks. Therefore, the binding energy peak near 725 eV in Figure 10b belongs to the characteristic peak of Fe 2p1/2 rather than Cs 3d. Due to the insufficient adsorption capacity of α-FeOOH for Cs^+^, the characteristic peak of Cs 3d cannot be observed. Generally, XPS test results are more accurate when the mass content of elements in the sample exceeds 5%. In this case, the low adsorption capacity of α-FeOOH for Sr^2+^, Cs^+^ and I^−^ leads to the absence of characteristic peaks.

## 4. Conclusions

In this study, the typical metal tank corrosion product α-FeOOH was proposed as an adsorbent for the absorption of Co^2+^, Ni^2+^, Sr^2+^, Cs^+^ and I^−^. The influences of external conditions on the properties of adsorbent was systematically studied, and the adsorption mechanism was elucidated. The adsorption equilibrium for Co^2+^, Ni^2+^, Sr^2+^, Cs^+^ and I^−^ occurred at 72 h, 48 h, 24 h, 24 h, and 12 h, respectively. The adsorption kinetic of Co^2+^, Ni^2+^ and I^−^ is more consistent with that of chemisorption. The morphology of nuclides and the positive and negative charges on α-FeOOH change with the increasing pH values in the system. The adsorption of Co^2+^, Ni^2+^ and Sr^2+^ showed the best efficiency under alkaline conditions. With increasing pH, the adsorption capacity of Cs^+^ initially increases and then decreases, while the adsorption capacity of I^−^ gradually decreases. The adsorption capacity of α-FeOOH for Co^2+^, Ni^2+^, Sr^2+^ and I^−^ increases with increasing temperature. The calculation of thermodynamic parameters reveals that the adsorption behavior of the aforementioned four nuclides involves entropy increase and heat absorption. In contrast, Cs^+^ exhibits entropy decrease and heat release, which corresponds to the experimental observation of decreased adsorption capacity with increasing temperature. The calculation of thermodynamic parameters reveals that the adsorption behavior of the aforementioned four nuclides involves entropy increase and heat absorption. In contrast, Cs^+^ exhibits entropy decrease and heat release, which corresponds to the experimental observation of decreased adsorption capacity with increasing temperature. Due to the competitive coordination between negative ions, cation ions in the electrolyte, and nuclide ions, the adsorption capacity of Sr^2+^, Cs^+^ and I^−^ decreases with increasing ionic strength of the system. XPS patterns were used to analyze the main characteristic peaks before and after α-FeOOH adsorption, the peak of 854.4 eV was consistent with the binding energy of Ni-O bond, indicating that Ni^2+^ interacts strongly with oxygen on the surface of α-FeOOH.

## Figures and Tables

**Table 1:** Calculation of isotope ion reserve solution amount for preparation.

Nuclide Ions	Inorganic Salts	M_ion_ (g∙mol^−1^)	M_inorganic salts_ (g∙mol^−1^)	Calculated Dosage (mg)
Co^2+^	Co(NO_3_)_2_·6H_2_O	58.9	291.03	370.5815
Ni^2+^	Ni(NO_3_)_2_·6H_2_O	58.69	290.79	371.6008
Sr^2+^	Sr(NO_3_)_2_	87.63	211.63	181.1280
Cs^+^	CsNO_3_	132.9	194.91	109.9944
I^−^	KI	126.9	166.0	98.2636

Note: M_ion_—Molecular weight of ion; M_inorganic salts_—Molecular weight of inorganic salts.

**Table 2:** Adsorption experimental conditions.

Research Object	Experimental Conditions	Main Selection Condition
Adsorption time	1 min, 10 min, 30 min, 1 h, 3 h, 6 h, 12 h, 18 h, 24 h, 36 h, 48 h, 72 h	72 h
pH	2.0~12.0	6.5~7.5
Temperature	20 °C, 28 °C, 36 °C, 44 °C, 52 °C	28 °C
Ionic strength (NaClO_4_)	0 mmol/L, 0.5 mmol/L, 1 mmol/L, 5 mmol/L, 10 mmol/L, 50 mmol/L, 100 mmol/L	0 mmol/L

**Table 3:** At pH 6.5~7.5, temperature 28 °C and NaClO_4_ 0 mmol/L, the α-FeOOH of nuclide ions adsorption equilibrium data.

Nuclide Ions	Adsorption Equilibrium Time (h)	*q_e_* (mg∙g^−1^)	*K_d_* (m^3^∙kg^−1^)
Co^2+^	48	0.69	0.196
Ni^2+^	72	0.78	0.247
Sr^2+^	24	0.2	0.044
Cs^+^	24	0.34	0.039
I^−^	12	0.029	0.031

Note: *q_e_*—Equilibrium adsorption capacity; *K_d_*—Distribution coefficient.

**Table 4:** The calculated parameters of the pseudo first-order and pseudo second-order kinetic models for sorption of Co^2+^, Ni^2+^ and I^−^.

Nuclide Ions	Pseudo-First-Order Kinetic Model			Pseudo-Second-Order Kinetic Model		
	k_1_ (min^−1^)	*q_e_*/(mg·g^−1^)	R^2^	k_2_/(g·mg^−1^·min^−1^)	*q_e_*/(mg·g^−1^)	R^2^
Co^2+^	0.0616	0.660	0.960	0.2135	0.674	0.978
Ni^2+^	0.0596	0.726	0.951	0.1831	0.743	0.971
I^−^	0.0145	0.0269	0.940	0.6605	0.0295	0.982

**Table 5:** α-FeOOH adsorption of Co^2+^ (R^2^ = 0.8044), Ni^2+^ (R^2^ = 0.8429), Sr^2+^ (R^2^ = 0.9282), Cs^+^ (R^2^ = 0.9544), I^−^ (R^2^ = 0.6276), the thermodynamic parameters.

Nuclide Ions	Δ*S* (J·mol^−1^·K^−1^)	Δ*H* (kJ·mol^−1^)	∆*G* (kJ·mol^−1^)				
			293.15 K	301.15 K	309.15 K	317.15 K	325.15 K
Co^2+^	133.442	25.966	−13.153	−14.220	−15.288	−16.356	−17.423
Ni^2+^	112.297	19.500	−13.420	−14.319	−15.217	−16.115	−17.014
Sr^2+^	151.091	31.683	−12.609	−13.818	−15.027	−16.236	−17.444
Cs^+^	−40.348	−19.432	−7.604	−7.281	−6.959	−6.636	−6.313
I^−^	95.839	21.411	−6.684	−7.451	−8.218	−8.984	−9.751

## Data Availability

The data presented in this study are available on request from the corresponding author due to privacy.
